# [Corrigendum] Highly sensitive detection of leukemia cells based on aptamer and quantum dots

**DOI:** 10.3892/or.2025.8962

**Published:** 2025-07-30

**Authors:** Yating Yu, Siliang Duan, Jian He, Wei Liang, Jing Su, Jianmeng Zhu, Nan Hu, Yongxiang Zhao, Xiaoling Lu

Oncol Rep 36: 886–892, 2016; DOI: 10.3892/or.2016.4866

Following the publication of the above article, the authors drew to the Editor's attention that, concerning the histological images shown in Fig. 5B on p. 890, two pairs of the data panels showed overlapping sections, such that these data were derived from the same original source where the panels were intended to show the results from differently performed experiments.

Upon examining their original data, the authors realized that inadvertent errors were made in assembling the data in this figure. The corrected version of Fig. 5, now showing replacement data for the liver and lung images in Fig. 5B, is shown on the next page. Note that this error did not affect the overall conclusions reported in the paper. All the authors agree with the publication of this corrigendum, and are grateful to the Editor of *Oncology Reports* for allowing them the opportunity to publish this. They also apologize to the readership for any inconvenience caused.

## Figures and Tables

**Figure 5.**
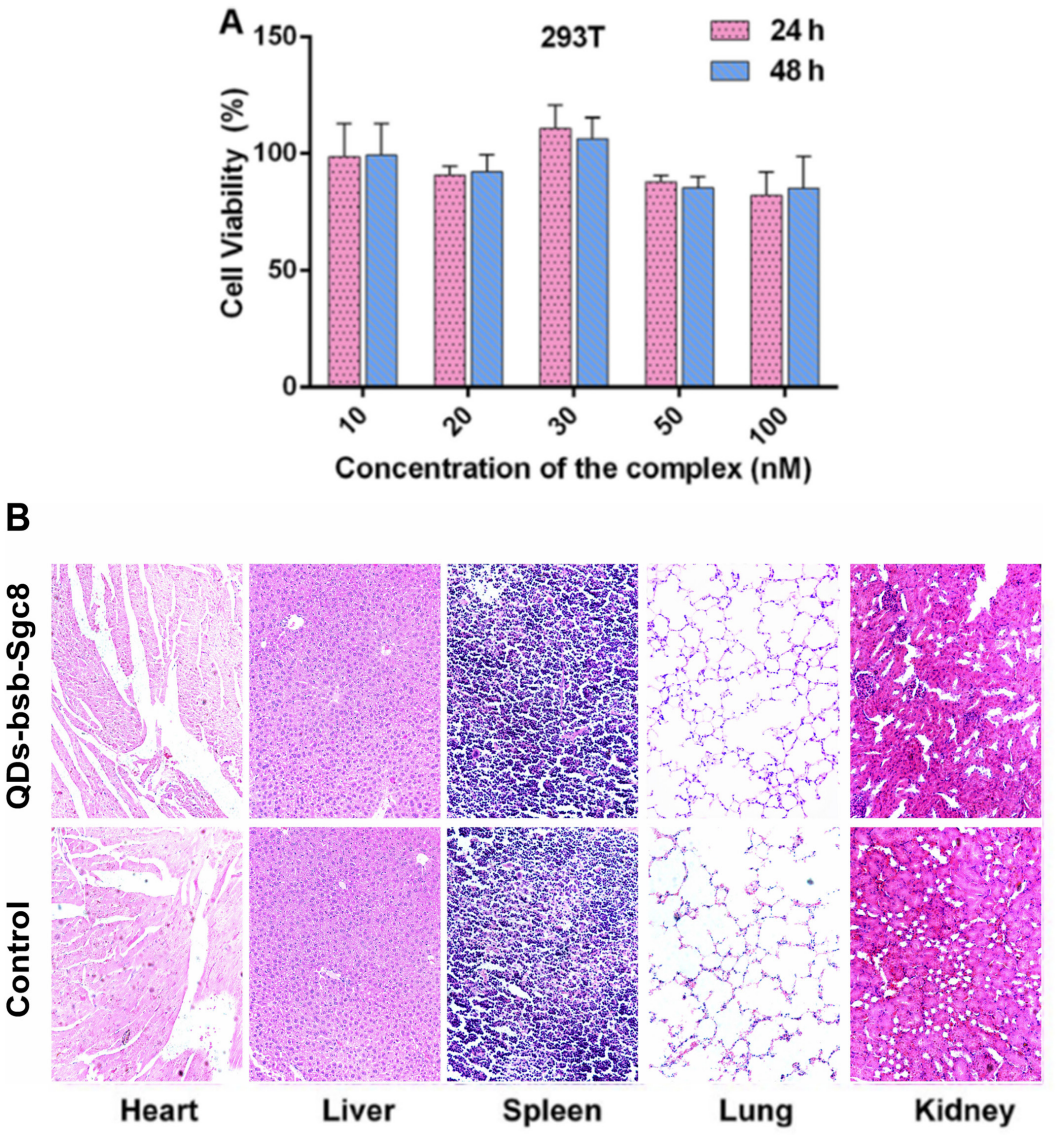
Non-toxicity of the QDs-bsb-apt complex to 293T normal cells (A) and organs such as the heart, liver, spleen, lung and kidney (B). 293T cells were incubated with various concentrations of the QDs-bsb-Sgc8 complex (10, 20, 30, 50 and 100 nM) for 24 h (red column) or 48 h (blue column). (B) Comparative safety assessment of QDs-bsb-Sgc8 compared with the PBS group 20 days following a single intravenous administration in nude mice (n=3). No histologic evidence of toxicity in any organ was identified.

